# Central-marginal dynamics in *Dinoponera quadriceps* (Hymenoptera, Formicidae): activity density, body reduction and functional divergence

**DOI:** 10.1007/s00114-026-02124-0

**Published:** 2026-06-08

**Authors:** Sabrina Medeiros, Bruno Mayrink, Jhonathan Silva, Ricardo Campos, Tatiana Cornelissen, Marcilio Fagundes

**Affiliations:** 1 Insecta: Center for Insect Biology and Taxonomy, DBG/CCBS, Universidade Estadual de Montes Claros, Montes Claros, Minas Gerais Brazil; 2 Universidade Federal do Vale do Rio São Francisco, Tomaz Guimarães, Senhor do Bonfim, Bahia Brazil; 3 Universidade Federal de Viçosa, Viçosa, Minas Gerais Brazil; 4 Center for Ecological Synthesis and Conservation, Universidade Federal de Minas Gerais, Belo Horizonte, Minas Gerais Brazil

**Keywords:** Ant ecology, Geographic range limits, Phenotypic variation, Species distribution, Trait-based ecology

## Abstract

The Central-marginal hypothesis predicts that populations occurring at the periphery of a species’ geographic distribution experience more adverse environmental conditions, resulting in reduced population density, lower fitness, and potential morphological changes. In insects, morphological traits are strongly associated with ecological performance and resource acquisition, making them useful indicators of how populations respond to environmental gradients. Here, we investigated whether populations of the ant *Dinoponera quadriceps* differ in activity density and morphofunctional traits between the center and edge of the species’ geographic distribution along the Espinhaço Mountain Range, Brazil. Ants were sampled using pitfall traps in two sites approximately 610 km apart. Generalized Linear Mixed Models were used to evaluate differences in activity density and trait variation between sites, and a Principal Component Analysis summarized multivariate body size variation. The activity density of *D. quadriceps* was -higher in the central population in the full dataset but this difference was not robust to the removal of a single outlier trap. A positive correlation between D. quadriceps activity density and richness of other ant species was observed in the full dataset but also disappeared after outlier exclusion. Individuals from the marginal population exhibited significantly smaller overall body size. Additionally, trait-specific differences emerged, with marginal individuals displaying larger cephalic index, longer femora, and larger eyes. These findings suggest that peripheral environments impose energetic constraints that reduce body size while favoring morphological adjustments that enhance locomotor and sensory efficiency, highlighting the importance of intraspecific functional variation in understanding species responses at geographic range limits.

**Supplementary Information:**

The online version contains supplementary material available at 10.1007/s00114-026-02124-0.

## Introduction

A central tenet in biogeography is that a species’ geographic distribution is shaped by its capacity to maintain viable populations across varying environmental gradients (Sexton et al. 2009). According to the central–marginal hypothesis, peripheral populations are often exposed to more adverse or suboptimal environmental conditions compared to central populations, which typically occupy more stable and favorable habitats (see Brown et al. 1996; Pironon et al. 2017). Consequently, central populations are expected to exhibit higher densities and superior performance, whereas edge populations may experience demographic decline and reduced fitness (Holt and Barfield 2011; Eriksson and Rafajlovic 2022). Environmental and demographic shifts at range margins often drive significant changes in morphological patterns, including reduced trait variability and smaller body size due to energetic constraints, stabilizing selection, and intensified genetic drift (Kirkpatrick and Barton 1997; Arnett and Gotelli 1999; Braz et al. 2023). Furthermore, the occupation of suboptimal habitats may reinforce such differences, favoring phenotypes with greater metabolic efficiency under stressful conditions, typical of range limits (Pyron 1999; Economo et al. 2015).

In social insects such as ants, morphological traits are closely tied to ecological performance and niche breadth (Guilherme et al. 2019). The interplay between environmental filters and morphological adaptation is particularly evident in traits related to resource acquisition and locomotion (see Wahl et al. 2015). For instance, mandible and clypeus size, as well as head length and width, are associated with prey capture, manipulation, and processing capacity, in addition to reflecting bite force and feeding efficiency (Gronenberg 1995; Paul 2001; Larabee et al. 2017). Similarly, eye width is related to visual perception and foraging activity (Esquivel et al. 2017), whereas antennal scape length is linked to sensory efficiency and environmental exploration (Elgar et al. 2018). Metafemur length is associated with locomotion and displacement capacity during foraging (Sommer and Wehner 2012), while Weber’s length is a classical indicator of body size, often correlated with metabolic costs, dispersal ability, and tolerance to environmental stressors (Abeikova et al. 2022). Importantly, an integrated analysis of these morphofunctional traits allows for a robust assessment of the functional performance and adaptive potential of populations subjected to distinct environmental pressures at the limits of their distribution (Gronenberg 1995; Sexton et al. 2009; Sommer and Wehner 2012; Larabee et al. 2017).

The Neotropical ant *Dinoponera quadriceps* (Formicidae: Ponerinae) provides an ideal model for testing these dynamics because it combines a distinctive social structure, characterized by the absence of morphologically differentiated queens, with a broad distribution across environmentally contrasting habitats (Medeiros and Araújo 2014; Vieira et al. 2024). This omnivore ant (Medeiros et al. 2012) is found in the Caatinga, Cerrado, and Atlantic forest formations, occurring throughout much of northeastern Brazil (Batista et al. 2021). This broad distribution reflects its adaptation to environments characterized by high temperatures and pronounced climatic seasonality (Araújo and Rodrigues 2006; Medeiros and Araújo 2014). Beyond its wide range, the species exhibits a distinctive social structure in which a single fertile worker (gamergate) assumes the reproductive role (Monnin and Peeters 1998). Foraging is solitary and guided by chemical and spatial cues, with workers showing high fidelity to specific paths while hunting for a generalist diet (Azevedo et al. 2021; Vieira et al. 2024). The combination of solitary foraging and a broad geographic reach suggests that morphological traits related to resource acquisition and manipulation are under strong selective pressure across varying environmental conditions (Guilherme et al. 2019). Thus, it is reasonable to expect differentiation of morphological traits among populations in response to local conditions, potentially enhancing energetic efficiency during foraging in distinct habitats.

In addition to evaluating the focal species, we also quantified the richness of co-occurring ant species at each site as an indirect indicator of local community context and potential biotic pressure. Ant species richness may influence resource availability, competitive interactions, and niche occupation, all of which can affect the performance and persistence of focal populations, particularly near distributional limits (Economo et al. 2015; Eriksson and Rafajlovic 2022). Under the central–marginal framework, we expected edge populations of *D. quadriceps* to occur in assemblages with lower species richness due to harsher environmental conditions and reduced habitat suitability toward range margins (Pironon et al. 2017; Eriksson and Rafajlovic 2022). Alternatively, if richness remained similar between regions, this would suggest that population-level responses of *D. quadriceps* are more strongly associated with species-specific physiological or morphological constraints than with broad community impoverishment.

In this study, we compared population density and morphofunctional traits of *D. quadriceps*, and local ant species richness between two populations located at the center and at the southern edge of the species’ geographic distribution. While most studies on range limits focus on community-level shifts in species presence or richness (Eckert et al.,^2008^; Pironon et al. 2017), there is a significant knowledge gap regarding how individuals within a single species adjust their phenotype to survive at the edge of their ecological tolerance. We addressed this by testing two premises of the central–marginal distribution hypothesis: (i) populations located at the center of the species’ distribution exhibit higher activity density, as environmental resources are expected to be more suitable and predictable in central areas (Eriksson and Rafajlovic 2022); (ii) edge populations consist of individuals with smaller body size compared to those from central populations, due to greater energetic constraints at the range limits (Economo et al. 2015). In addition, we predicted that peripheral sites would support lower richness of co-occurring ant species, reflecting stronger environmental constraints toward the range margins and generating a positive relationship between *D. quadriceps* abundance and the richness of other ant species. Finally, by moving beyond simple body size, we hypothesized that individuals at the margins would exhibit shifts in multivariate trait composition potentially associated with locomotor and sensory functions, alongside greater among-individual variation.

## Materials and methods

### Study area and site characterization

**Fig. 1:**
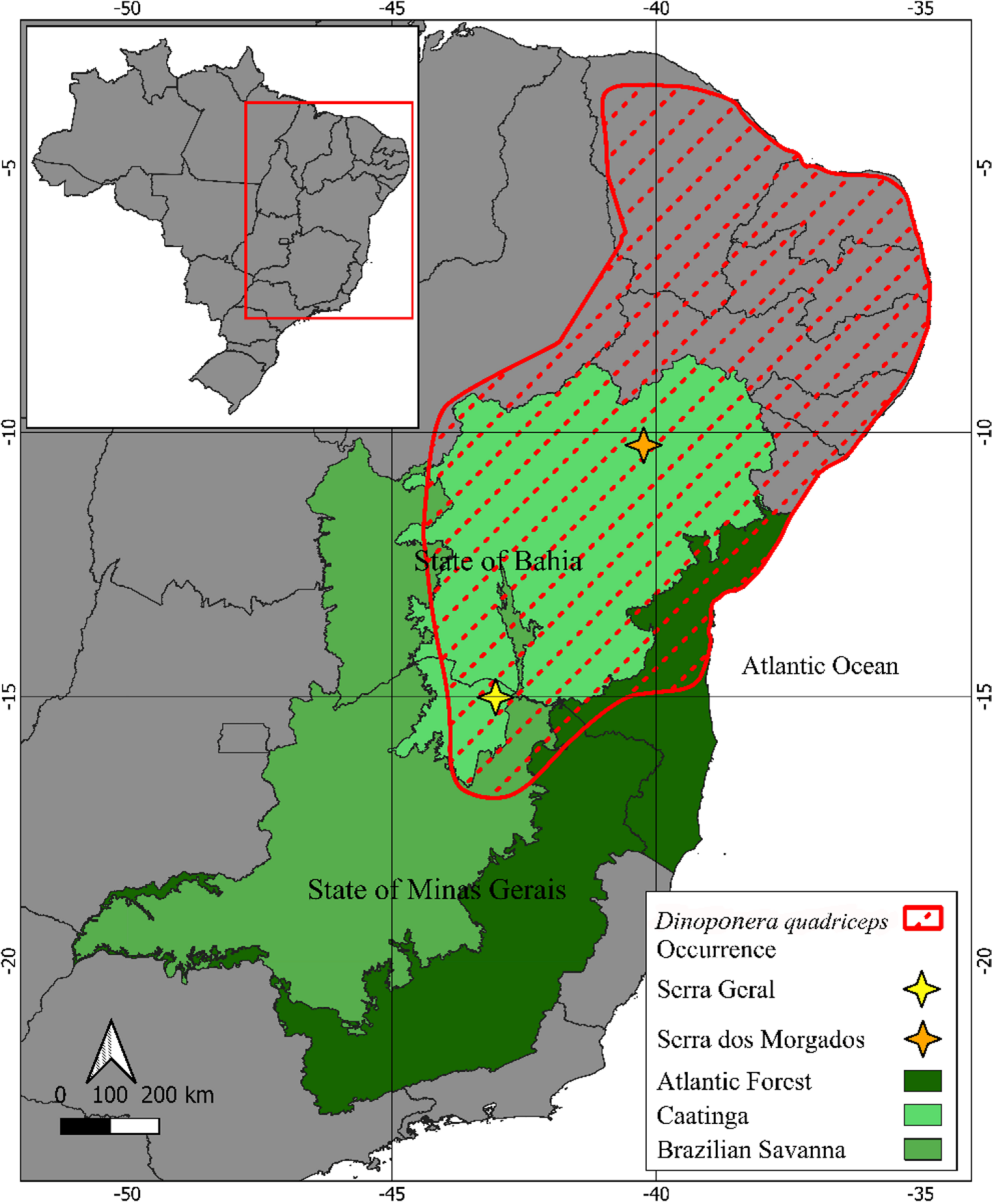
Map showing the sampling sites and the known geographic distribution of *Dinoponera quadriceps*, based on Paiva and Brandão (1995) and occurrence records obtained from GBIF (GBIF.org 2025; download generated in December 2025)

The populations of *Dinoponera quadriceps* targeted in this study were located in Serra Geral (15°00′ 27″ S and 43°00′ 49″ W) and Serra dos Morgados (10°14′42″ S and 40°14′06″ W), both situated within the Espinhaço Range. The Espinhaço Range extends for over 1,200 km across the states of Minas Gerais and Bahia, forming one of the most ancient and biologically unique landscapes in Brazil (Fernandes et al. 2016, Oswald et al. 2025). The two sampling sites (Serra Geral and Serra dos Morgados) are approximately 610 km apart and are situated at elevations of 890 m and 960 m above sea level, respectively. The climate at both sites is markedly seasonal, with a dry and cooler period extending from April to September and a warm and rainy season from October to March. At Serra Geral, the mean annual temperature is approximately 24 °C with mean annual precipitation of 830 mm (Fagundes et al. 2022). Serra dos Morgados presents a slightly warmer and drier climate, with a mean annual temperature of 26 °C and annual precipitation of approximately 650 mm (Cavalcanti et al., 2006). The predominant vegetation type at both sites is Seasonal Deciduous Forest, characterized by the dominance of plant species that lose at least 50% of their leaves during the prolonged dry season (Quesada et al. 2009). Based on the known geographic distribution of *D. quadriceps* and the relative location of the sampling sites relative to the species’ range limits, the population from Serra dos Morgados was classified as ‘central’, whereas the population from Serra Geral was classified as ‘marginal’ (Fig. 1). Although each distributional condition was represented by a single sampling site, both areas share similar vegetation structure and seasonality, allowing a comparative assessment of populations occurring under contrasting positions within the species’ geographic range. Nonetheless, some local environmental characteristics may also contribute to the observed patterns and should be considered when interpreting the results.

### Ant sampling

Ant sampling was carried out during the 2024 rainy season. At each site, three 900 m² plots (30 × 30 m), spaced at least 150 m apart from each other. In each plot, five pitfall traps were buried at ground level at the vertices and the center, totaling 30 traps across both sites. The traps consisted of a 1 L plastic containers filled with 500 mL of a preservative solution (10 mL neutral detergent, 490 mL water, and 10 g NaCl). The traps remained in the field for 48 h. Each pitfall trap was sampled only once during the study period. Sampled ants were stored in 70% ethanol and transported to the INSECTA collection (Center for Biology and Insect Taxonomy at Universidade Estadual de Montes Claros, Unimontes). Specimens were sorted and identified using the taxonomic keys of Baccaro et al. (2015) and Feitosa et al. (2024), supplemented by the AntWeb database and consultation with specialists.

### Morphofunctional trait measurements

**Fig. 2:**
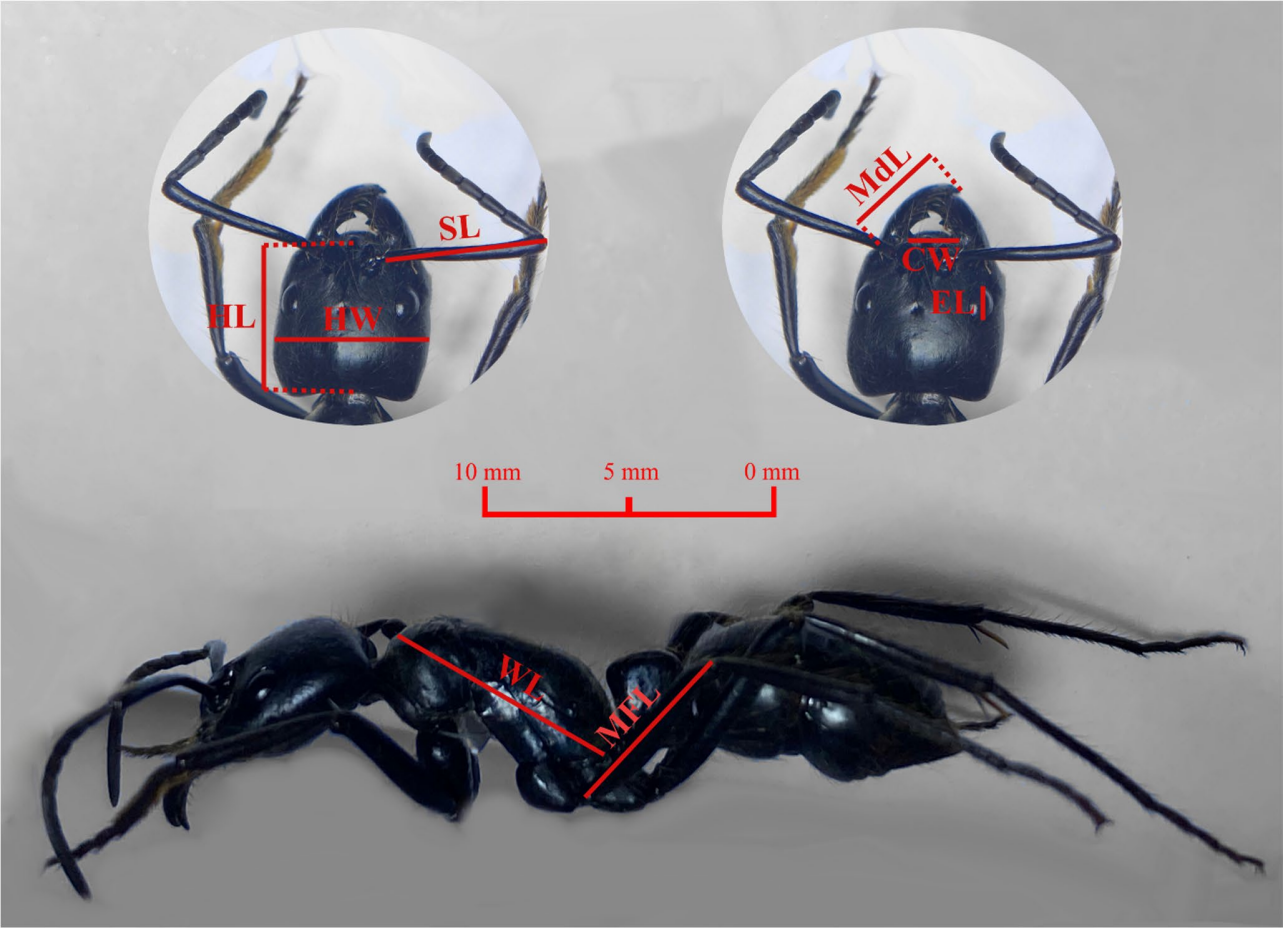
Morphofunctional traits measured in *Dinoponera quadriceps*: (MdL) mandible length, (EL) eye length, (CW) clypeus width, (HL) head length, (HW) head width, (SL) antennal scape length, (WL) Weber’s length and, (MFL) metafemur length

A total of 36 individuals of *D. quadriceps* (18 individuals per site) were used to characterize morphofunctional traits. This number of individuals were used since the fewest number of ant individuals to capture accurate means of trait variation at the species level is six (Gaudard et al. 2019). Individuals were obtained from different pitfall traps within each plot, reducing the likelihood that all measured workers originated from the same colony. However, because nests were not directly tracked or sampled, colony identity could not be determined, and some degree of non-independence among individuals cannot be completely excluded. We measured head length (HL), head width (HW), Weber’s length (WL), clypeus width (CW), antennal scape length (SL), eye length (EL), metafemur length (MFL), and mandible length (MdL) (Fig. 2). The cephalic index (CI) was calculated as CI = HW/HL x 100. These structures were selected as they represent functional adaptations to environmental conditions, specifically resource acquisition, locomotion and sensory perception (Kaspari and Weiser 2002; Davidson et al. 2004; Weiser and Kaspari 2006; Bihn et al. 2010). All measurements were performed using a ZEISS Stemi 305 stereomicroscope equipped with an Axiocam 208 color camera and ZEISS ZEN (ver. 3.2) imaging software.

### Statistical analysis

Generalized Linear Mixed Models (GLMMs) were used to evaluate the effects of site location (central vs. marginal) and local ant richness (excluding *D. quadriceps*) species on the abundance of *D. quadriceps* (*n* = 30 traps). Ant species richness was calculated as the total number of ant species recorded per pitfall trap, excluding *D. quadriceps*. Because pitfall traps estimate the number of active workers captured rather than true colony density, the number of individuals recorded was interpreted as a proxy for activity density throughout the analyses. In these models, the abundance of *D. quadriceps* was considered the response variable, while the collection sites, the species richness of other ants, and the interaction between site and species richness were considered explanatory variables. Thus, the model structure included site and ant richness as fixed effects, plot identity as a random effect, and a Poisson error distribution with log-link function. The Model significance was tested with Type II Wald chi-square using the Anova function of the car package in R version 4.5.2 (R Development Core Team 2025). Residual analyses were used to verify the adequacy of all models using the ‘DHARMa’ R package (Hartig 2016). Because only three plots were available per site, the random effect was included primarily to account for potential non-independence among traps within plots rather than to provide a robust estimation of variance components. Therefore, inferences regarding plot-level variation should be interpreted cautiously. In addition, because each distributional condition (central and marginal) was represented by a single site, site-specific environmental characteristics may also contribute to the observed patterns.

To assess whether overall body size differed between central and marginal populations, first we ordinated the individuals from both populations using a Principal Component Analysis (PCA) based on the correlation matrix of seven morphofunctional traits measured for the 36 individuals. Subsequently, scores from the first PCA axis (PC1) were compared using a GLM in which the collection site was used as the explanatory variable and PC1 scores (assumed to follow a Gaussian distribution) were the response variable. This served as a proxy for multi-trait body size variation. Although the number of individuals analyzed is within the range commonly used in ant trait-based studies, the relatively small sample size may reduce the statistical power to detect subtle morphofunctional differences among populations. Therefore, non-significant results should be interpreted cautiously.

To distinguish overall body size variation from size-independent shape variation, Weber’s length (WL) was first analyzed separately as a direct proxy for body size. Subsequently, the remaining morphofunctional traits [clypeus width (CW), antennal scape length (SL), eye length (EL), metafemur length (MFL), mandible length (MdL), cephalic index (CI)] were standardized by WL prior to analysis in order to evaluate proportional trait variation independent of overall body size (Oliveira et al. 2022). For the PCA, we included the raw (non-standardized) trait measurements to allow body size to emerge as the primary axis of variation. WL was retained in the PCA as a separate variable to confirm its loading on PC1. This approach is descriptive and does not affect the separate analysis of standardized traits. Separate GLMs were then used to compare each standardized trait between central and marginal populations. The significance of these models was assessed using ANOVA and F-test. At the end of all analyses, residuals were carried out to verify the adequacy of the error distribution used. Residual analyses were obtained using the ‘rdiagnostic’ function of the ‘RT4Bio’ R package (Reis-Jr et al. 2015). All statistical analyses were conducted in R version 4.5.2 (R Development Core Team 2025).

## Results

**Fig. 3:**
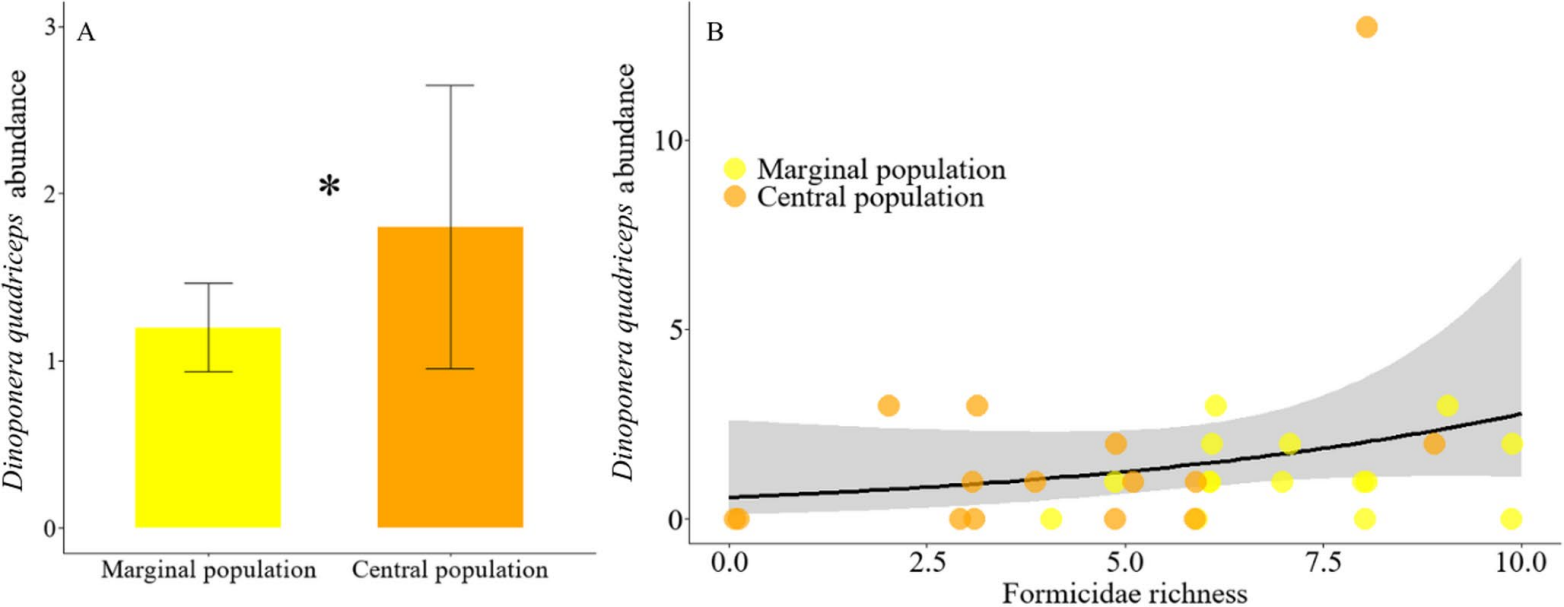
(**A**)Variation in the abundance of *Dinoponera quadriceps* (mean ± SE) between marginal and central populations, and (**B**) the relationship between *D. quadriceps* abundance and the richness of other ant species. The asterisk in figure (**A**) denotes a significant difference in mean abundance between populations (*p* < 0.05), and the shaded area in figure (**B**) indicates the 95% confidence interval

A total of 45 individuals of *D. quadriceps* were sampled across the two study sites (central = 27; marginal = 18). The mean ant abundance per pitfall trap varied significantly as a function of sampling site and the richness of other ant species (Table 1). Specifically, the mean abundance of *D. quadriceps* was 50% higher in the central population than in the marginal population (Fig. 3A). In addition, *D. quadriceps* abundance was positively related with the richness of other ant species (Fig. 3B).

**Table 1:** Results of the Generalized Linear Mixed Model (GLMM) evaluating the effects of site, other co-occurring ant species richness and, the interactions between these variables on the abundance (activity density) of *Dinoponera quadriceps*

Response Variable	Explanatory Variable	Estimate	Std. Error	z	X^2^	*P*
Abundance	Site	1.055	0.398	2.649	10.46	**0.0081**
	Richness	0.260	0.082	3.182	13.88	**< 0.001**
	Interaction	0.252	0.159	1.578	2.4886	0.114

Because one pitfall trap in the central population captured an exceptionally high number of *D. quadriceps* individuals (see Fig. 3B), we conducted a sensitivity analysis excluding this outlier. After its removal, the GLMM showed no significant difference in activity density between sites and no relationship with ant richness (see Supplementary Material 1).

**Fig. 4:**
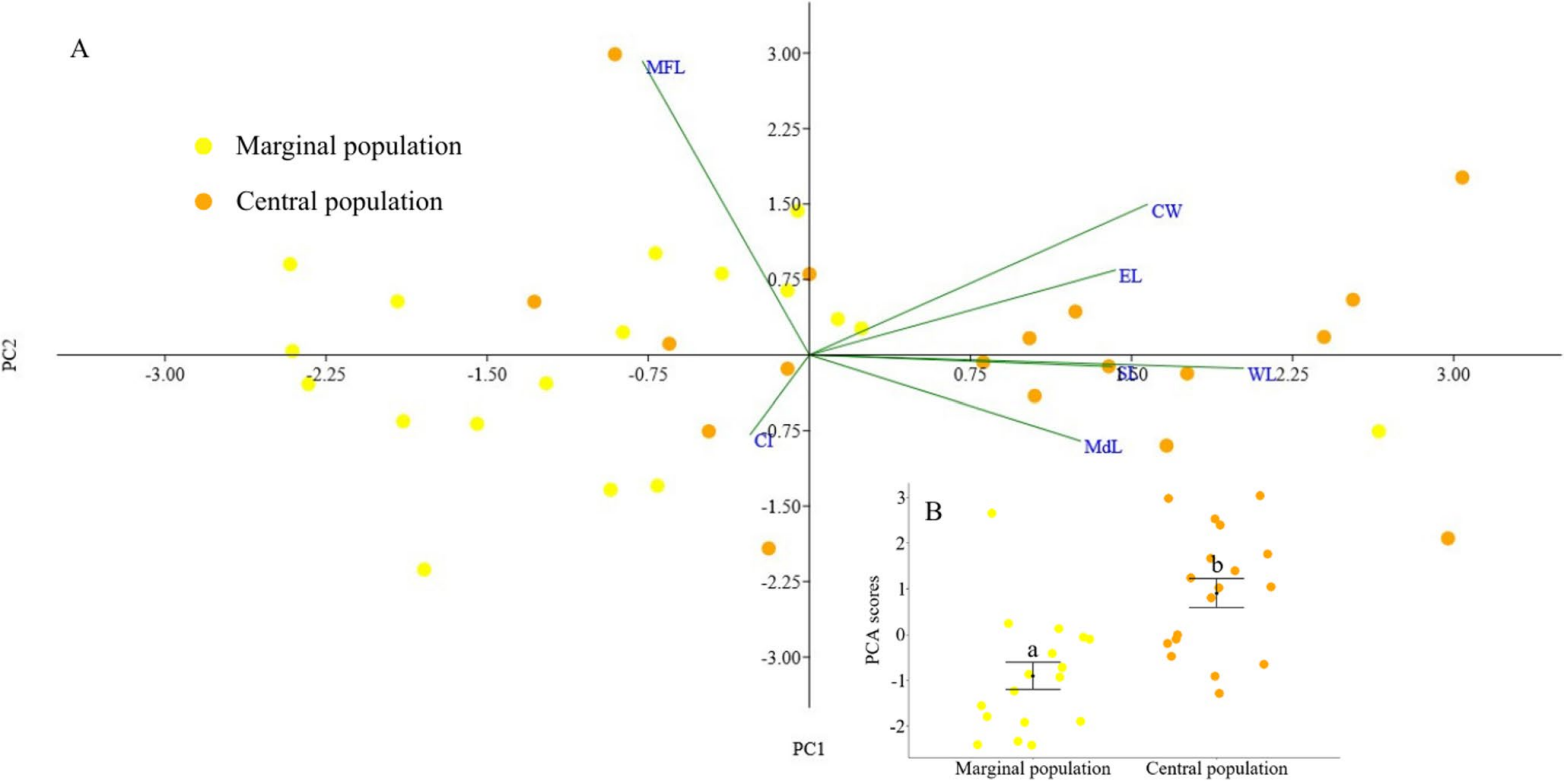
Principal Component Analysis (PCA) based on morphofunctional traits (MFL: metafemur length, CW: clypeus width, EL: eye length, SL: antennal scape length, WL: Weber length, MdL: mandible length, CI: cephalic index) of *Dinoponera quadriceps* individuals from central and marginal populations, showing: (**A**) a biplot ordering individual ants along the first two PCA axes, along with the loadings of morphofunctional traits; and (**B**) the statistical variation in first principal component scores between marginal and central populations. Arrow length reflects the magnitude of each trait’s contribution, and direction indicates correlation with the principal components

The first two PCA axes accounted for 50.7% of the total variation in the size of the morphofunctional traits of *D. quadriceps* (PC1 = 35.5%; PC2 = 15.2%). Overall, central population clustered positively along PC1, showing strong associations with WL, CW, SL, EL, and MdL, whereas the marginal population was negatively associated with this axis (Table 2; Fig. 4A). Given that measurements of the main morphofunctional traits were used in the PCA, it is reasonable to assume that the first axis likely represents overall body size in *D. quadriceps*. Accordingly, PC1 scores were significantly higher in the central population than in the marginal population (Deviance = 973.89, F = 12.088, *P* = 0.0014; Fig. 4B), indicating that individuals from the central population exhibit a larger overall body size than those from the range margin.

**Table 2:** Loadings of morphofunctional traits on the first two principal components (PC1 and PC2) from the Principal Component Analysis (PCA) of *Dinoponera quadriceps* individuals

Trait*	PC1 (35.5%)	PC2 (13.2%)
Weber Length (WL)	0.56366	−0.036984
Eye Length (EL)	0.39759	0.2353
Scape Length (SL)	0.39347	−0.032306
Clypeus Width (CW)	0.43893	0.41741
Mandible Length (MdL)	0.35197	−0.23751
Metafemur Length (MFL)	−0.21671	0.81438
Cephalic Index (CI)	−0.076714	−0.21991

PC1 explains 35.5% of the total variance, while PC2 explains 13.2%. Loading values indicate the magnitude and direction of each trait’s contribution to the principal components. Measurements used in the PCA were non-standardized

**Fig. 5:**
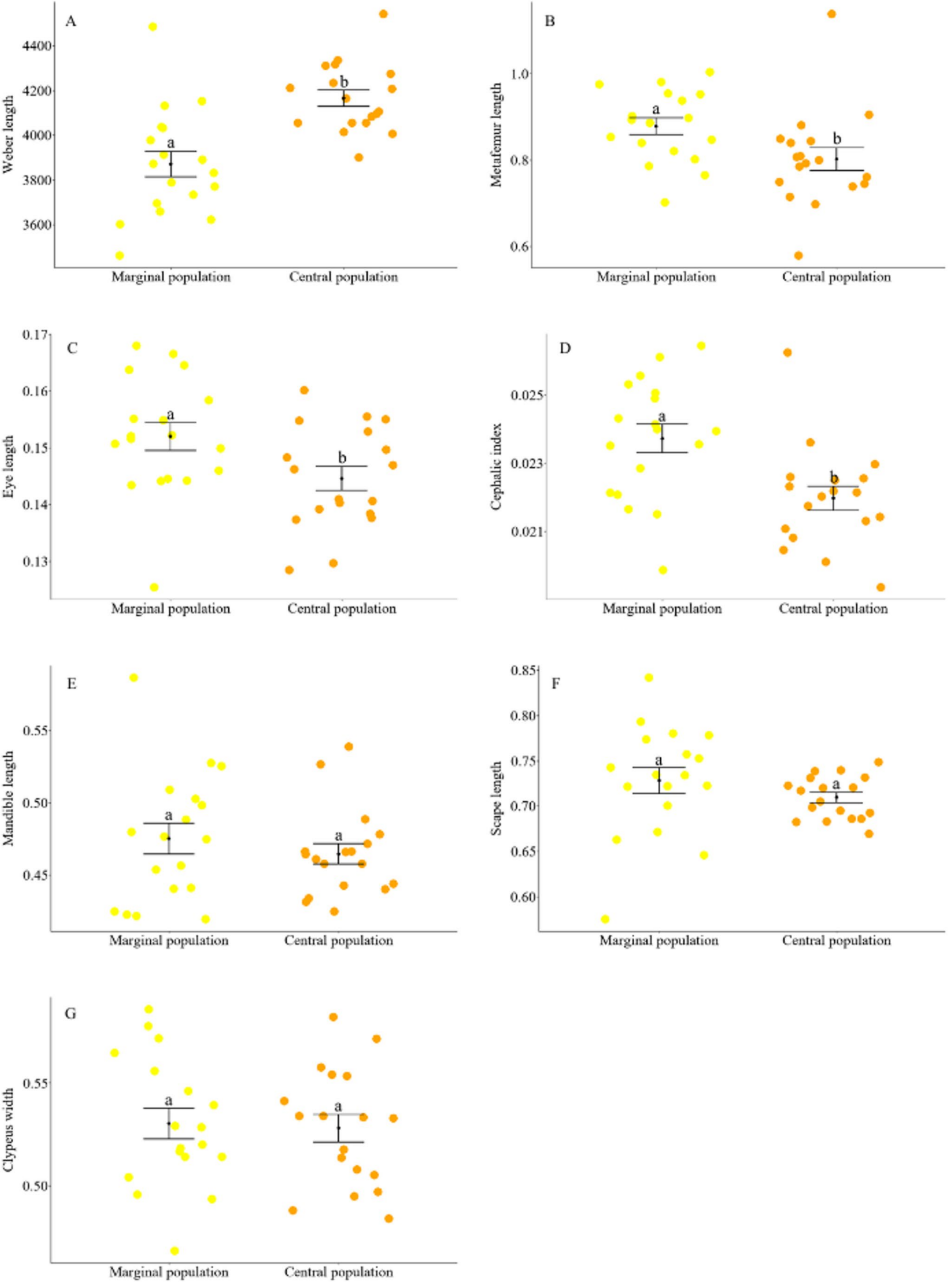
Morphofunctional traits variation between marginal and central populations. (**A**) Weber’s length, (**B**) metafemur length, (**C**) eye length, (**D**) cephalic index, (**E**) scape length, (**F**) mesosoma length, and (**G**) clypeus width (*n* = 36)

Analysis of individual traits revealed specific morphological divergences between sites. Individuals from the central population exhibited a significantly greater Weber’s length than those from the marginal population (Table 3; Fig. 5A). Conversely, individuals from the central population displayed a lower cephalic index, shorter metafemur length, and shorter eye length compared to individuals from the marginal population (Fig. 5B, C, D). The other morphofunctional traits, such as mandible length, scape length and, clypeus width did not differ significantly between the two populations (Table 3; Fig. 5E, F, G).

**Table 3:** Results of the Generalized Linear Model (GLM) evaluating the effects of sites (marginal or central) on the standardized morphofunctional traits of *Dinoponera quadriceps*

Response Variable	Estimate	Std. Error	F	*P*
Weber length	294.580	67.850	18.848	**0.0001**
Metafemur length	0.075	0.033	5.281	**0.0278**
Eye length	−0.007	0.003	5.216	**0.0287**
Cephalic index	−0.002	0.001	10.252	**0.0029**
Mandible length	−0.011	0.013	0.670	0.4186
Scape length	−0.019	0.016	1.490	0.2306
Clypeus width	−0.002	0.010	0.049	0.8254

## Discussion

Our results are broadly consistent with the central-marginal hypothesis, as we observed higher density of *Dinoponera quadriceps* in the central site, and individuals from the marginal population exhibited a significant reduction in body size. However, these findings should be interpreted with caution because our study design included only a single site representing each distributional condition. As a consequence, local environmental differences between Serra Geral and Serra dos Morgados cannot be fully disentangled from effects associated with central versus marginal range position. Therefore, the observed patterns should not be interpreted as definitive evidence of the central–marginal hypothesis, but rather as site-level patterns that are compatible with its predictions. Here it is important highlight that ant worker abundance was estimated based on the number of individuals captured in pitfall traps. However, pitfall captures represent a proxy for worker activity outside the nest rather than absolute colony number (Vasconcellos et al. 2004; Segev et al. 2015; Maák et al. 2020). In addition, worker activity in *D. quadriceps* is strongly influenced by local climatic conditions, with the number of workers captured per pitfall negatively correlated with temperature (Medeiros et al. 2012). Given that Serra dos Morgados experiences higher temperatures than Serra Geral (Fagundes et al. 2022; Cavalcanti et al., 2006), differences in activity patterns between sites may partially influence the observed abundance patterns. Therefore, the higher activity density recorded in the central population should be interpreted cautiously and may not directly reflect differences in colony density. In this context, the number of sampling units and the spatial scope of the study should be considered when extrapolating these patterns, particularly given potential local heterogeneity in ant activity and resource distribution. Notwithstanding these considerations, the consistency and direction of the observed differences between central and marginal populations.

Regardless of the sampling site, we observed a positive relationship between the abundance of *D. quadriceps* and the richness of other ant species. Importantly, this relationship does not necessarily indicate direct ecological interactions between *D. quadriceps* and the remaining ant assemblage. Instead, the positive association may reflect shared environmental drivers such as habitat heterogeneity and local resource availability, which may simultaneously promote higher ant richness and increased activity of *D. quadriceps* (see Neves et al. 2013; Kuchenbecker et al., 2018; Deák et al. 2021). Structurally complex environments can provide a wider range of nesting sites, thermal refuges, and food resources, thereby supporting both community diversity and ant activity (Kovalenko et al. 2012). Therefore, the reduced activity density observed in the marginal population may reflect not only processes associated with range position, but also broader environmental differences influencing the entire ant community at Serra Geral. Finally, it is crucial to point out that our models lost significance after we removed an outlier showing no difference in mean abundance *D. quadriceps* between sites and no relationship between ant abundance and the richness of other ant species. However, these results must be taken with caution, as this outlier might be influenced by the proximity of one *D. quadriceps* colony. Given the known bias of pitfall traps placed near nests (Vasconcellos et al. 2004), we interpret the outlier-removed analysis as more biologically meaningful. Therefore, our data do not provide robust support for the abundance-related prediction of the central–marginal hypothesis.

The PCA and Weber’s length comparisons indicated that individuals of *D. quadriceps* from the central population exhibited larger overall body size. Although PC1 was used as a proxy for body size, this interpretation assumes that all traits loaded positively on this axis and should be considered an approximation of multivariate size variation rather than a direct measure. The reduction in body size at the margin is consistent with expectations of energetic and developmental constraints imposed by suboptimal habitats (e.g., Economo et al. 2015; Braz et al. 2023). In insects, body size is strongly influenced by resource availability during development; smaller phenotypes often emerge when energetic intake is limited (Brown, Stephens and Kaufman, 1996; Holt and Barfield 2011). Our findings suggest that marginal *D. quadriceps* populations may be operating closer to their physiological and energetic limits, leading to the observed ‘morphological reduction’. Because we did not directly quantify prey biomass, food availability, or larval nutritional conditions, the mechanisms underlying this reduction remain speculative. *Dinoponera quadriceps* is a solitary omnivorous predator whose workers forage individually for arthropods and other food resources (Medeiros et al. 2012; Azevedo et al. 2021). Therefore, variation in local prey availability or foraging conditions between sites may indirectly affect larval nutrition and adult body size. In addition, Serra Geral presents a slightly cooler and wetter climate than Serra dos Morgados, conditions that may alter worker activity, prey dynamics, and colony energetic balance. Cooler temperatures may reduce foraging efficiency and resource acquisition rates, while higher humidity may influence prey composition and availability in the leaf litter. Such environmental differences could affect larval growth and developmental investment, ultimately contributing to the smaller body size observed in the marginal population.

Moreover, the observed morphofunctional differences may also reflect non-adaptive processes, including phenotypic plasticity associated with local environmental conditions or stochastic processes such as genetic drift in geographically peripheral populations. Because colony identity was not determined, we cannot fully exclude the possibility that some sampled individuals belonged to the same nest. Although individuals were collected from multiple traps and plots, this limitation should be considered when interpreting fine-scale morphofunctional variation. Furthermore, the relatively small number of individuals analyzed may reduce the statistical power to detect subtle trait differences, particularly for non-significant comparisons. However, even with this sampling limitation, we still have found some strong differences between sites (e.g., *D. quadriceps* from the central population exhibited much bigger Weber’s length, Fig. 3).

Crucially, we found that morphological responses were trait-specific rather than uniform. Individuals from the marginal population exhibited a larger cephalic index, longer femora, and greater eye length, whereas mandible, antennal scape, and clypeus width did not differ. This pattern suggests that peripheral environments may influence the combination of morphofunctional traits expressed by individuals. Because most traits were standardized by Weber’s length, these differences primarily reflect variation in shape rather than absolute size, indicating potential shifts in proportional investment among traits. The relatively larger head (cephalic index) and eyes observed in the marginal population may be consistent with differences in sensory-related traits in the more open, heterogeneous environment typical of range limits, while longer femora may be associated with differences in locomotor performance or exploratory behavior (Wahl et al. 2015; Sommer and Wehner 2012). In a solitary forager like *D. quadriceps* (Azevedo et al. 2014, 2021), these trait differences could potentially influence how individuals explore the environment and locate spatially dispersed prey. In contrast, the stability of mandibles and clypeus length may indicate stronger functional constraints on traits directly related to prey capture and chemical communication, which are functionally non-negotiable for this species’ ecology. Finally, we must emphasize that all those functional interpretations related to locomotion, sensory and predatory efficiency fo *D. quadriceps* are hypotheses rather than directly tested adaptive responses.

In summary, our study demonstrates that the effects of range margins on species may not be fully captured by activity-based abundance metrics alone, but are also reflected in patterns of intraspecific morphological variation. We provide evidence that *D. quadriceps* exhibits reduced overall body size at the range margin alongside shifts in trait proportions, i.e. to lower metabolic costs while also differing in proportional investment in traits related to locomotion and sensory perception, although the underlying mechanisms (e.g., adaptive responses, plasticity, or demographic processes) remain unresolved. Nevertheless, because only one central and one marginal site were evaluated, our results should be interpreted as preliminary evidence consistent with central–marginal expectations rather than broad support for the hypothesis across the species’ distribution. Additional studies including multiple populations across the geographic range will be necessary to disentangle site-specific environmental effects from general range-position effects. Importantly, while the morphofunctional differences between populations were robust and unaffected by outlier exclusion, the abundance-related patterns were not. This dissociation suggests that morphological responses to range margins may be more sensitive indicators of environmental stress than activity-based metrics, which can be strongly influenced by local sampling artifacts.

This study highlights why ant functional ecology must move beyond simple activity-based metrics; intraspecific variation can reveal the hidden stress of marginal populations long before a demographic reduction occurs. As climate change continues to shift environmental conditions creating new gradients, understanding these morphofunctional adjustments will be critical for predicting the persistence of apex invertebrate predators at their geographic boundaries. Future studies integrating environmental measurements, behavioral data, and experimental approaches will be essential to determine whether the observed trait variation translates into differences in ecological performance or fitness.

## Supplementary Information

Below is the link to the electronic supplementary material.

Supplementary File 1 (DOCX 15.8 KB)

## Data Availability

No datasets were generated or analysed during the current study.

## References

[CR1] Abeikova L, Boudinot BE, Beutel RG et al (2022) The skeletomuscular system of the mesossoma of *Formica rufa* workers (Hymenoptera: Formicidae). Insect Syst Divers 6:1–6. 10.1093/isd/ixac002

[CR2] Araújo A, Rodrigues Z (2006) Foraging behavior of the queenless ant *Dinoponera quadriceps* Santschi (Hymenoptera: Formicidae). Neotrop Entomol 35:159–164. 10.1590/S1519-566X2006000200002 17348125 10.1590/s1519-566x2006000200002

[CR3] Arnett AE, Gotelli NJ (1999) Bergmann’s rule in ant lion *Mymeleon immaculatus* DeGeer (Neuroptera: Myrmeleontidae): geographic variation in body size and heterozygosity. J Biogeogr 26:275–283

[CR4] Azevedo DLO, Medeiros JC, Araújo A (2014) Adjustments in the time, distance and direction of foraging in *Dinoponera quadriceps* workers. J Insect Behav 27:177–191. 10.1007/s10905-013-9412-6

[CR5] Azevedo DLO, Medeiros JC, Araújo A (2021) Flexibility in the integration of environmental information by *Dinoponera quadriceps* during foraging. Rev Bras Entomol 65:e20210084. 10.1590/1806-9665-RBENT-2021-0084

[CR6] Baccaro FB, Feitosa RM, Fernández F, Fernandes IO, Izzo T, Souza JLP, Solar R (2015) Guia para os gêneros de formigas do Brasil. Editora INPA, Brasil. 10.5281/zenodo.32912

[CR7] Batista T, Nascimento IC, Carneiro MAF, Bernardo CSS, Saha A, Carvalho KS (2021) Association of *Dinoponera quadriceps* nests with termite mounds and landscape variables in the Caatinga dry forest, Brazil. Insectes Soc 68:41–47. 10.1007/s00040-020-00806-0

[CR8] Bihn JH, Gebauer G, Brandl R (2010) Loss of functional diversity of ant assemblages in secondary tropical forests. Ecology 91:782–782. 10.1890/08-1276.1 20426336 10.1890/08-1276.1

[CR9] Braz AG, Figueiredo MS, Weber MM, Grelle CEV (2023) Morphological variability decreases in populations living in less suitable environments and close to the range edges. J Biogeogr 50:1749–1762. 10.1111/jbi.14687

[CR10] Brown JH, Stevens GC, Kaufman DM (1996) The geographic range: size, shape, boundaries and internal structure. Annu Rev Ecol Syst 27:597–623. 10.1146/annurev.ecolsys.27.1.597

[CR11] Cavalcanti NB, Resende GM (2006) Ocorrência de xilopódio em plantas nativas de imbuzeiro. Rev Caatinga 19:287–293

[CR12] Davidson DW, Cook SC, Snelling RR (2004) Liquid-feeding performances of ants (Formicidae): ecological and evolutionary implications. Oecologia 139:255–266. 10.1007/s00442-004-1508-4 15034777 10.1007/s00442-004-1508-4

[CR13] Deák B, Báthori F, Lorinczi G et al (2021) Functional composition of ant assemblages in habitat islands is driven by habitat factors and landscape composition. Sci Rep 11:e20962. 10.1038/s41598-021-00385-5 10.1038/s41598-021-00385-5 PMC8546063 34697323

[CR14] Eckert CG, Samis KE, Lougheed SC (2008) Genetic variation across species’ geographical ranges: the central-marginal hypothesis and beyond. Mol Ecol 17:1170–1188. 10.1111/j.1365-294X.2007.03659.x 18302683 10.1111/j.1365-294X.2007.03659.x

[CR15] Economo EP, Sarnat EM, Janda M et al (2015) Breaking out of biogeographical modules: range expansion and taxon cycles in the hyperdiverse ant genus *Pheidole*. J Biogeogr 42:2289–2301. 10.1111/jbi.12592 27660394 10.1111/jbi.12592 PMC5014176

[CR16] Elgar MA, Zhang D, Wang Q et al (2018) Insect antennal morphology: the evolution of diverse solutions to odorant perception. Yale J Biol Med 91:457–469 30588211 PMC6302626

[CR17] Eriksson M, Rafajlovic M (2022) The role of phenotypic plasticity in the establishment of range margins. Philos Trans R Soc Lond B Biol Sci 377:e20210012. 10.1098/rstb.2021.0012 10.1098/rstb.2021.0012 PMC8784930 35067091

[CR18] Esquivel FR, Leitner N, Zeil J, Narendra A (2017) The sensory arrays of the ant, *Temnothorax rugatulus*. Arthropod Struct Dev 46:552–563. 10.1016/j.asd.2017.03.005 28347859 10.1016/j.asd.2017.03.005

[CR19] Fagundes M, Silva APMF, Mayrink BHS et al (2022) Seed germination of a myrmecochorous plant endemic to the Brazilian semiarid region. Acta Bot Bras 36:e20220093. 10.1590/1677-941X-ABB-2022-0093

[CR20] Feitosa RM, Dias AM (2024) An illustred guide for the identification of ant subfamilies and genera in Brazil. Insect Syst Evol 55:451–571. 10.1163/1876312X-bja10062

[CR51] Fernandes GW (2016) The megadiverse rupestrian grassland. In: Fernandes GW (ed) Ecology and Conservation of Mountaintop Grasslands in Brazil. Springer, Cham, pp 3-14

[CR21] Gaudard CA, Robertson MP, Bishop TR (2019) Low levels of intraspecific trait variation in a keystone invertebrate group. Oecologia 190:725–735. 10.1007/s00442-2019-04426-9 31172253 10.1007/s00442-019-04426-9 PMC6704090

[CR22] GBIF.org (2025) GBIF Occurrence Download. 10.15468/dl.c23pev

[CR23] Gronenberg W (1995) The fast mandible strike in the trap-jaw ant Odontomachus: I. Temporal properties and morphological characteristics. J Comp Physiol A Neuroethol Sens Neural Behav Physiol 176:391–398. 10.1007/BF00219064

[CR24] Guilherme DR, Souza JLP, Franklin E et al (2019) Can environmental complexity predict functional trait composition of ground-dwelling ant assemblages? A test across the Amazon Basin. Acta Oecol 99:e103434. 10.1016/j.actao.2019.05.004

[CR25] Hartig H (2016) DHARMa: residual diagnostics for hierarchical (Multi-Level/Mixed) regression models. R package, version 0.1.2. https://github.com/florianhartig/ DHARM (accessed 02 September 2025)

[CR26] Holt RD, Barfield M (2011) Theoretical perspectives on the statics and dynamics of species’ borders in patchy environments. Am Nat 178:6–25. 10.1086/661784 10.1086/661784 PMC5014989 21956092

[CR27] Kaspari M, Weiser MD (2002) The size-grain hypothesis and interspecific scaling in ants. Funct Ecol 13:530–538. 10.1046/j.1365-2435.1999.00343.x

[CR29] Kirkpatrick M, Barton NH (1997) Evolution of a species’ range. Am Nat 150:1–23. 10.1086/286054 18811273 10.1086/286054

[CR30] Kovalenko KE, Thomaz SM, Warfe DM (2012) Habitat complexity: approaches and future directions. Hydrobiologia 685:1–17. 10.1007/s10750-011-0974-z

[CR31] Kuchenbecker J, Fagundes M (2018) Diversity of insects associated with two common plants in the Brazilian Cerrado. Eur J Entomol 115:354–363. 10.14411/eje.2018.035

[CR32] Larabee FJ, Gronenberg W, Suarez AV (2017) Performance, morphology and control of power-amplified mandibles in the trap-jaw ant *Mymoteras*. J Exp Biol 220:3062–3071. 10.1242/jeb.156513 28855320 10.1242/jeb.156513

[CR33] Maák I, Trigos-Peral G, Slipnski P, Grzes IM, Horváth G, Witek M (2020) Habitat features and colony characteristics influencing ant personality and its fitness consequences. Behav Ecol 32:124–137. 10.1093/beheco/araa112 33708007 10.1093/beheco/araa112 PMC7937185

[CR34] Medeiros J, Araújo A (2014) Workers’ extra-nest behavioral changes during colony fission in *Dinoponera quadriceps* (Santschi). Neotrop Entomol 43:115–121. 10.1007/s13744-013-0193-6 27193517 10.1007/s13744-013-0193-6

[CR35] Medeiros J, Araújo A, Araújo HFP, Queiroz JPC, Vasconcellos A (2012) Seasonal activity of *Dinoponera quadriceps* Santschi (Formicidae, Ponerinae) in the semi-arid Caatinga of northeastern Brazil. Rev Brasil de Entomol 56:81–85. 10.1590/S0085-56262012000100013

[CR36] Monnin T, Peeters C (1998) Monogyny and regulation of reproduction in the queenless ant *Dinoponera quadriceps*. Anim Behav 55:299–306. 10.1006/anbe.1997.0601 9480697 10.1006/anbe.1997.0601

[CR37] Neves F, Queiroz-Dantas K, DaRocha W, Delabie JHC (2013) Ants of Three Adjacent Habitats of a Transition Region Between the Cerrado and Caating Biomes: The Effects of Heterogeneity and Variation in Canopy Cover. Neotrop Entomol 42:258–268. 10.1007/s13744-013-0123-7 23949808 10.1007/s13744-013-0123-7

[CR52] Oliveira FMP, Costa FVC, Lopes CT et al (2022) Morphology of four common and phylogenetically distant ant speciesvaries along disturbance and aridity gradients in the Caatinga dry forest. Biotropica 54:78–90. 10.1111/btp.13029

[CR53] Oswald CB, Silveira FAO, Cornelissen T et al (2025) Biogeography and evolution of the vertebrate fauna in campo rupestre, a megadiverse Neotropical montane open ecosystem. Biol J Linn Soc 145:blaf032. 10.1093/biolinnean/blaf032

[CR38] Paiva RVS, Brandão CRF (1995) Nests, worker population, and reproductive status of workers, in the giant queenless ponerine ant *Dinoponera* Roger (Hymenoptera: Formicidae. Ethol Ecol Evol 7:297–312. 10.1080/08927014.1995.9522938

[CR39] Paul J (2001) Mandible movements in ants. Comp Biochem Physiol Mol Integr Physiol 131:7–20. 10.1016/S1095-6433(01)00458-5 10.1016/s1095-6433(01)00458-5 11733162

[CR40] Pironon S, Papuga G, Villellas J, Angert AL, García MB, Thompson JD (2017) Geographic variation in genetic and demographic performance: new insights from an old biogeographical paradigm. Biol Rev Camb Philos Soc 92:1877–1909. 10.1111/brv.12313 27891813 10.1111/brv.12313

[CR41] Pyron M (1999) Relationships between geographical range size, body size, local abundance and habitat breadth in North American suckers and sunfishes. J Biogeogr 26:549–558. 10.1046/j.1365-2699.1999.00303.x

[CR54] Quesada M, Sanchez-Azofeifa GA, Alvarez-Añorve M et al (2009) Succession and management of tropical dry forestsin the Americas: review and new perspectives. Ecol Manag 258:1014–1024. 10.1016/j.foreco.2009.06.023

[CR42] R Development Core Team (2025) R: A language and environment for statistical computing. The R Foundation for Statistical Computing, Vienna Austria

[CR43] Reis-Jr R, Oliveira ML, Borges GRA (2015) RT4Bio: R tools for biologists. Available at: https://sourceforge.net/projects/rt4bio

[CR44] Segev U, Kigel J, Lubin Y, Tielborger K (2015) Ant abundance along a productivity gradient. PLoS One 10:e0131314. 10.1371/journal.pone.0131314 26176853 10.1371/journal.pone.0131314 PMC4503676

[CR45] Sexton JP, McIntyre PJ, Angert AL, Rice KJ (2009) Evolution and ecology of species range limits. Annu Rev Ecol Evol Syst 40:415–436. 10.1146/annurev.ecolsys.110308.120317

[CR46] Sommer S, Wehner R (2012) Leg allometry in ants: extreme long-leggedness in thermophilic species. Arthropod Struct Dev 41:71–77. 10.1016/j.asd.2011.08.002 21992805 10.1016/j.asd.2011.08.002

[CR47] Vasconcellos A, Santana GG, Souza AK (2004) Nest spacing and architecture of *Dinoponera quadriceps*. Braz J Biol 64:357–362. 10.1590/S1519-69842004000200022 15462310 10.1590/s1519-69842004000200022

[CR48] Vieira MEL, Teseo S, Azevedo DLO, Châline N, Araújo A (2024) Competition through ritualized aggressive interactions between sympatric colonies in solitary foraging ants. Sci Nat 111. 10.1007/s00114-024-01891-y 10.1007/s00114-024-01891-y 38289402

[CR49] Wahl V, Pfeffer S, Wittlinger M (2015) Walking and running in the desert ant *Cataglyphis fortis*. J Comp Physiol A 201:645–656. 10.1007/s00359-015-0999-2 10.1007/s00359-015-0999-2 PMC4439428 25829304

[CR50] Weiser MD, Kaspari M (2006) Ecological morphospace of New World ants. Ecol Entomol 31:131–142. 10.1111/j.0307-6946.2006.00759.x

