# Supplementary material for: Central-marginal dynamics in Dinoponera quadriceps (Hymenoptera, Formicidae): activity density, body reduction and functional divergence
Source: Naturwissenschaften. 2026 Jun 8;113(4):71. doi: 10.1007/s00114-026-02124-0 (PMC13246570; doi:10.1007/s00114-026-02124-0)
Supplement: Supplementary file 1 — (DOCX 15.8 KB) [file 114_2026_2124_MOESM1_ESM.docx]

**Table 1** Results of the Generalized Linear Mixed Model (GLMM) evaluating the effects of site, other ant species richness and, the interactions between these variables on the abundance of *Dinoponera quadriceps*.

| Response Variable | Explanatory Variable | Estimate | Std. Error | z | X^2^ | P |
| --- | --- | --- | --- | --- | --- | --- |
| Abundance | Site | -0.031 | 1.172 | -0.027 | 0.0305 | 0.8613 |
|  | Richness | 0.073 | 0.138 | 0.529 | 0.9163 | 0.3384 |
|  | Interaction | 0.018 | 0.178 | 0.099 | 0.0098 | 0.9211 |
